# Public Knowledge, Attitude, and Perception towards COVID-19 Vaccination in Saudi Arabia

**DOI:** 10.3390/ijerph181910081

**Published:** 2021-09-25

**Authors:** Salman Mohammed Al-Zalfawi, Syed Imam Rabbani, Syed Mohammed Basheeruddin Asdaq, Abdulhakeem S. Alamri, Walaa F. Alsanie, Majid Alhomrani, Yahya Mohzari, Ahmed A. Alrashed, Abdulaziz H. AlRifdah, Thabet Almagrabe

**Affiliations:** 1 College of Pharmacy, Qassim University, Buraydah 51452, Saudi Arabia; mhospital1920@gmail.com; 2 Department of Pharmacology and Toxicology, College of Pharmacy, Qassim University, Buraydah 51452, Saudi Arabia; syedrabbani09@yahoo.com; 3 Department of Pharmacy Practice, College of Pharmacy, AlMaarefa University, Dariyah, Riyadh 13713, Saudi Arabia; 4 Department of Clinical Laboratory Sciences, The Faculty of Applied Medical Sciences, Taif University, Taif 21974, Saudi Arabia; a.alamri@tu.edu.sa (A.S.A.); w.alsanie@tu.edu.sa (W.F.A.); m.alhomrani@tu.edu.sa (M.A.); 5 Centre of Biomedical Sciences Research (CBSR), Deanship of Scientific Research, Taif University, Taif 21974, Saudi Arabia; 6 Pharmacy Department, King Saud Medical City, Riyadh 12746, Saudi Arabia; yali2016@hotmail.com (Y.M.); a.alrifdah@ksmc.med.sa (A.H.A.); 7 Pharmaceutical Service Department, Main Hospital, King Fahad Medical City, Riyadh 11525, Saudi Arabia; aahalrashed@kfmc.med.sa; 8 Pharmaceutical Service Department, Dar Al Uloom University, Riyadh 13314, Saudi Arabia; emadfaiqa@gmail.com

**Keywords:** COVID-19 vaccine, vaccine hesitancy, knowledge, attitude, perception, perceived risk, Saudi Arabia

## Abstract

Coronavirus disease-19 (COVID-19) is a highly contagious infection that mainly affects the respiratory system of patients. To date, more than 10 million people have been affected by this virus, and Saudi Arabia has also reported over 210 million cases. At present, there is no established treatment for COVID-19. Vaccination is one of the ways to defeat the pandemic. Recent reports have indicated rare but serious adverse events after vaccination, causing an anxious response from the general public worldwide. Therefore, this study was aimed at evaluating the knowledge, attitude, and perception of the COVID-19 vaccine among the Saudi population. This study is a cross-sectional, web-based online survey conducted using a snowball sampling technique. A self-administered questionnaire prepared in Arabic and English was used to collect feedback from the general population on their knowledge, attitudes, and perceptions about the COVID-19 vaccine. Participants (*n* = 2022) from different regions of the country replied to the questions. The responses to the questions were recorded on a spreadsheet and analyzed using the SPSS software. Statistical analysis was performed using one-way ANOVA and non-parametric tests to draw conclusions about the results. Multivariate stepwise regression analysis was performed to determine the association between the knowledge, attitude, and perception scores and the demographic variables. *p* < 0.05 was used to indicate the significance of the data. The data from the study indicated that most of the participants were males (81%), between 18 and 59 years of age (85.9%), Saudi nationals (98.3%), and possessed graduation or above as a qualification (62.9%). The results suggest that a major portion of respondents have satisfactory knowledge (76%), a positive attitude (72.4%), and perception (71.3%) towards the use of COVID-19 vaccines. Their responses can be categorized as between ‘good’ and ‘fair’. However, 30–40% of respondents lacked information about COVID-19 vaccination availability for under 18-year-olds as well as for pregnant women, in addition to the lack of knowledge about the serious unreported adverse reactions and long-term protection offered by the vaccine against coronavirus. The correlation analysis between the variables (*p* > 0.05) indicated that the response to the KAP domains has no direct relationship. The survey results suggest that most of the Saudi population has sound knowledge and a positive attitude and perception. Since the COVID-19 vaccines have been approved for use in pregnancy and above 12-year-old children by health authorities, the lack of information shown by a significant percentage of participants requires strategies to update this information. Awareness programs targeting all sections of the population must be continued to provide all the updates, including vaccinations for pregnant women and children.

## 1. Introduction

COVID-19 is defined as a disease caused by the Severe Acute Respiratory Syndrome Coronavirus 2 (SARS-CoV-2). The illness has mostly affected patients who suffer from chronic comorbidities such as heart diseases, immunological defects, diabetes, and airway disease [1]. The disease is characterized by rapid transmission through the nasal route and can occur by close contact with an infected person [2]. The World Health Organization (WHO) in the month of January 2020 announced a public health emergency of international concern and called for a collaborative effort from all countries, including the Kingdom of Saudi Arabia (KSA), to contain the spread of the virus [3].

The important precautionary guidelines for COVID-19 recommended by the World Health Organization (WHO) include: wearing a facial mask, social distancing, and avoiding crowded and poorly ventilated places. These measures are strictly implemented by many countries. Largely, adherence to such precautionary practices depends on the behavior and is the social responsibility of the public [4]. Earlier studies indicated that the public’s knowledge and attitude towards a pandemic vary from region to region [5].

According to the literature, there is no treatment for COVID-19. Many interventions, such as corticosteroids and blood thinners, are being tried to minimize the complications of the disease [3]. Research is in progress in many centers to identify specific therapeutic agents that can effectively treat the infection [6]. Vaccination is suggested by WHO as the most suitable approach to build herd immunity in the population [1,4]. Currently, more than 100 COVID-19 vaccines are under various stages of development and testing. Though these vaccines are reported to be safe and can produce mild symptoms, in very rare cases, they have been associated with fatalities due to anaphylaxis and thrombotic events [7]. In the event of these unwanted adverse events and the lack of clear information on the duration of efficacy, many people still have apprehension about the COVID-19 jabs [8]. However, considering the pandemic situation and the benefit-risk ratio, WHO approved the use of vaccines for the general public, especially for the most vulnerable population, and several countries have started a mass inoculation program for their public [7].

The Kingdom of Saudi Arabia (KSA) is a vast country with a population of 34.81 million people. The country consists of 13 provinces and includes metropolitan cities, towns, and villages. The first positive case of COVID-19 was reported in the month of March 2020, and as of July 2021, KSA has recorded the second-highest number of coronavirus cases in the gulf region. The authorities have taken several proactive measures to control the spread of infection, including free vaccination for all people above 12 years of age [9].

Any new medical intervention has its own rate of acceptability among the public. Vaccine hesitancy is an important issue encountered among the population. Several factors such as public perception, communication, and media environment were reported to play a role in vaccine hesitancy [10]. The input from these resources was observed to affect the knowledge and attitude towards vaccines, hindering the mass vaccination programs [11]. In an earlier study, it was observed that 33% of US respondents showed hesitancy to taking the COVID-19 vaccine [12]. Similarly, around 31% of Turkish participants in an online survey indicated a refusal to be vaccinated against COVID-19 [13]. About 23% of Oman’s study participants in another study expressed concern over the safety of the COVID-19 vaccine [14]. Several factors were reported to influence the general population’s perception of the COVID-19 vaccine, such as adverse health consequences, lack of adequate knowledge about the safety and efficacy, long-term complications, and inadequate trust in the current health care system [15]. A study conducted in four major cities in Saudi Arabia suggested that the acceptability of the COVID-19 vaccine could be influenced by socio-demographic characteristics [16]. Analyzing people’s knowledge and other issues of perception based on facts and figures on practices is a scientific way for developing strategies that solve issues while also achieving the authorities’ desired objectives in society [17]. Hence, this study was planned to evaluate the knowledge, attitude, and perception of the COVID-19 vaccine among the Saudi population.

## 2. Materials and Methods

### 2.1. Study Setting and Population

This is a survey-based study conducted online among the Saudi population. People residing in the country, both males and females, from different educational levels and age groups, were involved in the study. A total of 2022 people responded to all the survey questions; hence, they were chosen for the study. The response to the survey was carefully selected to include participants from all the thirteen provinces of the country, such as Riyadh (12.2%), Makkah (10.1%), Eastern (10.3%), Al Madinah (7.8%), Al Qassim (7.2%), Hail (5.4%), Northern-border (6.4%), Asir (7.8%), Al Baha (6.9), Jazan (7.3%), Al Jawf (5.9%), Najran (6.8) and Tabuk (6.9). Respondents included people from different nationalities and professions but required internet access.

### 2.2. Ethical Clearance

The survey was conducted after ethical approval from the General Directorate of Health Affairs, Al-Qassim region, Registration number: 202103091 (IRB number: H-04-Q-001), dated 9 March 2021. Participants’ consent was taken while collecting the data for the study. When the participant clicked the link to the survey study, the informed consent appeared first, followed by the questionnaire. In the case of minors, the parents/guardians’ consent was collected before the data were analyzed. Participants were informed about the objective and purpose of the study in the informed consent. The participants were also informed that their identities would be kept anonymous, and the secrecy of their data was guaranteed.

### 2.3. Study Tool

The questionnaire was targeted at the general public using an online site popular for conducting survey studies. Snowball technology was adopted for conveying information to the public about the survey. Questions related to knowledge, attitude, and perception were utilized to record the response of the Saudi population to the COVID-19 vaccine.

### 2.4. Questionnaires Development and Validation

A questionnaire was developed in the English and Arabic languages. The expert committee constituted by the department validated the questionnaires. The committee members are comprised of professionals who have experience in survey analysis as well as proficiency in English and Arabic languages. The committee checked the content, language, suitability of questions for different domains, scoring patterns, etc. The pilot study included 25 carefully chosen participants of various ages, genders, professions, and educational qualifications. An expert committee evaluated their feedback and suggested necessary modifications before the questionnaire was posted online for data collection.

The members of the expert committee evaluated the face-validity and internal consistency of each question used for the domains of knowledge, attitude, and perception. After conducting the pilot study, the committee analyzed the responses to each question using Cronbach’s alpha reliability test [18]. Two questions from the perception domain (Q-3,5) scored an α value of 0.65, while one question from the knowledge domain (Q-3) scored α = 0.68, and one from the attitude domain obtained α = 0.69. The committee re-assessed these questions and modified them to match the internal consistency with the tested domains. Other questions used in the knowledge domain were found to score α value as Q-1 (0.81), Q-2 (0.83), Q-4 (0.80), and Q-5 (0.81). Similarly, the alpha value for other questions in the attitude domain was found to be Q-1 (0.80), Q-2 (0.82), Q-3 (0.80), and Q-5 (0.84), and Q-1 (0.82), Q-2 (0.85), and Q-4 (0.80) for the perception domain. The responses from the pilot study were not considered since some questions were found to be inconsistent, which were later modified before being used for online study.

The questionnaire was divided into two parts, namely, the socio-demographic section and the KAP section. In the socio-demographic part, respondents’ information such as gender, age, nationality, and educational qualifications was collected. While in the KAP, questions related to the knowledge contained a set of answers such as ‘Yes ‘, ‘No’ and ‘Don’t know’, while attitude and perception questions contained the answers ‘Agree’, ‘Disagree’ and ’Undecided’ [19].

The questions used for the knowledge domain were: do you have knowledge that Coronavirus infection can be prevented with a vaccine? (K-1); do you know that the COVID-19 vaccine is available free of cost after registering on the ‘Sahhaty’ App? (K-2); are you aware that the COVID-19 vaccine should be taken in two doses? (K-3); is it true that administration of the COVID-19 vaccine may cause mild side effects? (K-4); and is it correct that the COVID-19 vaccine is not recommended for people less than 18 years of age and pregnant women? (K-5). The attitude domain questions were: do you feel that the COVID-19 vaccine must be taken by all whenever it is available? (A-1); do you believe that the COVID-19 vaccine should be prioritized for a specific population, such as the elderly? (A-2); in your opinion, does the COVID-19 vaccine provide good protection after a few weeks against coronavirus infection? (A-3); in your understanding, does the COVID-19 vaccine cause any serious reactions apart from those that are reported? (A-4); and is it a moral obligation to discuss the benefits and risk factors associated with the COVID-19 vaccine with others? (A-5). The perception domain questions were: is it essential to vaccinate a large proportion of the population to defeat the coronavirus pandemic? (P-1); is it important to follow safety measures to avoid COVID-19 even after taking a vaccine? (P-2); do you think that the COVID-19 vaccine provides long-term protection against coronavirus infection? (P-3); do you prefer to take the COVID-19 vaccine if you are suffering from any health issues? (P-4); and do you prefer to take the COVID-19 vaccine if you are suffering from any health issues? (P-5).

### 2.5. Scoring Criteria

There were 15 questions in total, and each domain consisted of 5 questions. The scoring criteria were adopted from similar studies conducted in the past [14,20]. The correct answer to every question carried 1 mark, while a wrong or do not know/undecided carried 0 marks. This gave a total score range of 0–5 for knowledge, attitude, and perception domains. The average score for each demographic character was calculated, and if the score was above 3.5 (70%), then the response was categorized as ‘Good’, between 2.6–3.45 (51–69%) was categorized as ‘Fair’, and less than a 2.5 score (50%) was recorded as ‘Poor’.

### 2.6. Study Design

This was a cross-sectional questionnaire-based survey. The Saudi population residing in different regions of the kingdom took part in the online study. The survey was conducted in the months of March and April 2021. Responses received during the study period were screened. Participants responding to all the questions were further considered for analysis. A total of 2091 responses were received. However, 69 were rejected due to lack of complete information and, the percentage of rejection was found to be 3.29%. Among the rejected responses, the majority were secondary school qualified participants (64%), followed by high school (29%) and graduates (7%). The final analysis was performed for 2022 responses that contained complete information. Social media such as Twitter and WhatsApp were utilized for snowball technology in this study. The participants were requested to answer all the questions pertaining to three domains. Questions from each domain were displayed one after another, and a pop-up request appeared to answer the question before proceeding to the next question.

### 2.7. Inclusion and Exclusion Criteria

The general public residing in Saudi Arabia with access to the internet and who were willing to participate in the study were included. No personal request or reward was offered to participants to take part in the study. Incomplete filled responses to the survey questions were excluded.

### 2.8. Statistics

All completed survey forms were evaluated, and their responses were recorded in an excel spreadsheet. The categorical variables were represented as numbers and percentages. Continuous variables were mentioned as the mean scores with standard deviation. The data were analyzed, and statistical significance of the results between groups was performed through one-way ANOVA followed by a non-parametric post hoc test [21]. The non-parametric tests included a Spearman’s rho correlation, which was used to find the association between knowledge, attitude, and perception. The Mann–Whitney U test (one-sided) was used to compare scores of each domain with binary demographic groups: (Gender and Nationality). The Kruskal–Wallis H test (one-sided) was used to compare scores of each domain with demographic categories (Age and Qualification). The one-sided Chi-squared test was used to determine whether there was a significant association between the demographic variables. Additionally, descriptive univariant analysis of the data was performed by using the Mann–Whitney U test for the continuous variable, and the Chi-squared test was utilized for categorical variables. Furthermore, a multiple stepwise linear regression analysis was performed to determine the influence of demographic variables such as age, gender, nationality, and educational level on the total score of KAP as a dependent variable using SPSS-IBM 25 (IBM, Armonk, NY, USA). The data were compared and indicated as significant if the *p*-value was less than 0.05.

## 3. Results

### 3.1. Demographic Characteristics of the Participants

The demographic characteristics of the participants are represented in Table 1. Both male (81%) and female participants (19%) took part in the survey study, and the total number of respondents was 2022 from different regions of the country. In the age group category, the majority of the respondents were between the ages of 18 and 59 years (85.9%), followed by under 18 years (3.7%) and above 59 years (0.3%). Most of the participants were Saudi nationals (98.3%) and possessed educational qualifications of graduation and above (62.9%). In the educational category, the other groups were high school (32.1%) and secondary-school qualified respondents (5%).

### 3.2. Frequency of Responses

For the question that coronavirus infection can be prevented by a vaccine, about 82% of the respondents (both male and female) indicated ‘agreed’. The use of the ‘Sahhaty’ App for vaccine registration was ‘agreed’ by 93% of male and 95.3% of female participants. Regarding the vaccination regimen, 85.3% of male and 93.8% of female respondents ‘agreed’ that two doses need to be taken. A total of 70.8% of the male respondents ‘agreed’ that the COVID-19 vaccine could cause mild side effects, while 23.9% of males replied as ‘Don’t know’. However, in females, about 84% ‘agreed’ with the mild side effects after vaccination. Interestingly, 46.2% of males did not know that the COVID-19 vaccination is now recommended for children above 12 years of age as well as for pregnant women. Around 24.4% of females also indicated ‘Don’t know’ to this question, while 72.4% of them agreed that the COVID-19 vaccine is not preferred in this group of population (Table 2).

Table 3 represents the frequency of participants’ responses to the attitude questions. A total of 63.2% of males and 66.9% of females indicated a positive attitude towards taking the COVID-19 vaccine whenever it is available. Over 70% of respondents feel that the vaccine must be given in priority to a special group of the population, such as older-aged people. However, 39.5% of males and 36.7% of females ‘agreed’ that the COVID-19 vaccine provides good protection after a few weeks against coronavirus infections, where others did not show a similar attitude. Similarly, regarding the appearance of any serious reactions after COVID-19 vaccination, only 24.7% of males and 20.8% of females indicated ‘Yes’, whereas others did not have this attitude towards serious adverse reactions. Additionally, 64.7% of males and 72.4% of females felt a moral obligation to discuss the benefits and risk factors associated with the COVID-19 vaccine with others. Interestingly, for this question, 21.2% of males’ replies were ‘Don’t know’.

Positive feedback in the form of ‘yes’ (71.4% of males and 72.4% of females) was received for the question that vaccinating a large proportion of the population could defeat the coronavirus pandemic. Similarly, safety measures to be followed even after COVID-19 vaccination, a significant proportion of participants (75% males and 86.7% females) indicated a positive perception by answering ‘yes’. However, for the question of whether the COVID-19 vaccine provides long-term protection against coronavirus infection, only 41.7% of males and 33.3% of females ‘agree’, while others indicated either ‘no’ or ‘don’t know’. Moreover, 46.4% of males and 47.9% of females indicated ‘No’ to the question of whether they prefer to take the COVID-19 vaccine if they suffer from any health issues, whereas 34.7% of males and 33.9% of females replied ‘Yes’. Similarly, 39.5% of males and 40.1% of females did not feel that the COVID-19 vaccine could complicate the existing disease condition, while 37.8% of males and females replied ‘Yes’ to this question, and about 22% of respondents indicated a ‘Don’t know’ reply (Table 4).

### 3.3. Analysis of Participants’ Response

Analysis of the data indicated non-significant variation between different demographic characteristics for the knowledge, attitude, and perception domains. The mean ranking for the ‘knowledge’ score was found to be highest for female participants (4.29, *p* = 0.08), followed by graduates and above-qualified participants (3.92, *p* = 0.210), then between 18 and 59 years of age (3.90, *p* = 0.101) and Saudi nationals (3.85, *p* = 0.112), according to gender, education qualification, age, and nationality, respectively. In the ‘attitude’ questions, female participants scored high ranking (3.73, *p* = 0.289) in gender, 3.68 mean raking was observed for 18–59-year-old people (in age category), 3.89 for Saudi nationals (Nationality category) and graduates and above (3.70, *p* = 0.643). While in the ‘perception’ questionnaires, females (3.64, *p* = 0.653), 18–59-year-old people (3.62, *p* = 0.749), Saudis (3.62, *p* = 0.385) and graduation qualified participants have more perceptions (3.64, *p* = 0.778) about the issues related to COVID-19 (Table 5).

### 3.4. Categorization of Participants’ Score on KAP Domains

Table 6 represents the participants’ scores in the domain of KAP. In the knowledge domain, female participants had 83.1% good and 13.5% fair, while 64.7% of males had good and 22.2% had fair knowledge. In the age group category, the highest percentage of ‘good’ was found in people between 18 and 59 years (70.4), followed by people above 59 years (57.1%) and less than 18 years old (54.7%). In the nationality category, Saudis have 68.3% ‘good’ and 20.5% ‘fair’, while non-Saudis have 57.1% ‘good’ and 22.9% fair knowledge response. The data were found to be non-significant when a comparison was made. In the qualification, 71.4% of graduates, 63.4% of high school qualified, and 58.4% of secondary school qualified participants scored ‘good’, and the comparison within the group was found to be significant (*p* < 0.05).

For the attitude domain, 42.6% of males and 38% of females scored ‘good’. The comparison within this group was found to be significant (*p* < 0.05). However, other demographic characteristics showed non-significant variation. In the aged category, 18–59 years scored 51.7%, while below 18 years and above 60 years scored 41.7% and 42.9% ‘good’, respectively. In the nationality, Saudi citizens attained 41.8% and non-Saudis 37.1% ‘good’, while in the qualification category, the highest ‘good’ was recorded for graduates (48.8%), followed by secondary school (44.6%) and then high school (38.8%).

In the perception domain, male participants achieved 45%, and female participants received 43% of a ‘good’ score, while 27.8% of males and 37.2% of females scored a ‘fair’ response. People between 18 and 59 years received 54.5% ‘good’, less than 18 years received 45.7% and above 60 years received a 28.6% ‘good’ score. In the nationality, 48.6% of Saudi nationals received ‘good’ while non-Saudis received 44.5%. The ‘Good’ perception shown by different education categories were 45.6% graduation, 44.6% secondary school, and 42.8% high school qualified participants. None of the characteristics were found to be statistically significant.

### 3.5. Correlation between Various Domains of KAP

The correlation between various domains of KAP is summarized in Table 7. The Rho value for the variables; knowledge–attitude, was found to be 0.352 (*p* = 0.103), knowledge–perception was 0.301 (*p* = 0.332), and attitude–perception was 0.548 (*p* = 0.084).

### 3.6. Factors Affecting KAP on the Use of Vaccine

By keeping the total score of knowledge, attitude, and perception as the dependent variable, the multiple stepwise linear regression analysis was performed using demographic variables of the study such as age, gender, nationality, and educational level. As exhibited by Table 8, age and gender influenced the total KAP score to a significant extent. The educational level and nationality had no significant impact on the total KAP score.

## 4. Discussion

The present study evaluated the knowledge, attitude, and perception of the COVID-19 vaccine among the population of the Kingdom of Saudi Arabia. The population is comprised of either sex with different ages, nationalities, and educational backgrounds (Table 1). The observations from the study suggested that the population of Saudi Arabia that took part in the survey had ‘sound’ knowledge about the COVID-19 vaccine, its availability, its dosage, the possible side effects, and the non-recommended group of people for vaccination (Table 2).

Among the population, a non-significant mean knowledge ranking was found to be higher for female participants, people in the age group of 18–59 years, Saudi nationals, and graduation or above qualified people (Table 5). These groups of people also responded with more ‘good’ and ‘fair’ responses to the knowledge domain. However, when these groups were compared within themselves, except for educational qualification (*p* = 0.002), none of them were found to be significant (*p* > 0.05) (Table 6).

The findings are in agreement with two similar studies conducted in China and the United States. The studies suggested the ‘approval’ of newer prophylactic interventions such as vaccines for tackling the pandemic issues created by COVID-19. The data from these studies also indicated that gender, nationality, educational level, and occupation could have a positive influence on the knowledge domain of the participants [22,23]. One of the more noteworthy findings from this domain was that approximately 46% of males and 22% of females lacked the necessary knowledge regarding COVID-19 vaccinations for children under the age of 18 and pregnant women. The information has to be updated because the COVID-19 immunization is now approved in the Kingdom of Saudi Arabia for pregnant women and children over the age of 12 [24]. Strategies must be developed to convey all of the most recent updates on COVID-19 and the intervention methods used by health authorities to combat coronavirus infection.

In the ‘attitude’ domain, the study population of Saudi Arabia indicated a positive response to the COVID-19 vaccination, a preference for older people, and a moral obligation to discuss the benefits and risks with fellow citizens (Table 3). The mean ranking was found to be high among females, people of age 18–59 years, Saudi nationals, and graduate qualified participants (Table 5). Similarly, these groups of the population have scored higher percentages of either ‘good’ or ’fair’ attitudes. The comparison of the data within groups indicated that except for gender (*p* = 0.016), all other groups have non-significant (*p* > 0.05) variation (Table 6). The observations are in accordance with the previous survey conducted to find the acceptability of vaccination among the older generation population [25].

However, for questions on level of protection and appearance of serious unreported adverse reactions, a significant proportion (>40%) of respondents did not have proper information. The responses of the participants to these questions could be because the information about the duration of protection after COVID-19 vaccination is being updated regularly as the research in this area progresses (https://www.who.int/news-room/q-a-detail/coronavirus-disease-(COVID-19)-vaccines?adgroupsurvey={adgroupsurvey, accessed on 13 September 2021). Further, as the vaccination program has been expanded to several groups of the population, newer adverse events have been reported [26,27]. In an earlier study, the population of Russia (50%) and Poland (27%) indicated a greater degree of apprehension about possible unreported adverse reactions [28]. In this context, the response of the population (22% ‘Yes’) towards serious adverse reactions associated with the COVID-19 vaccine in this study (Table 3) has a logical meaning and is quite above the lowest reported values in other parts of the world.

In the ‘perception’ questionnaire, the contributors revealed a positive attitude towards defeating the coronavirus pandemic with vaccines, adhering to safety measures even after vaccination, and preference for vaccines over existing disease conditions (Table 4). The mean ranking values again suggested that females, people of age 18–59 years, Saudi nationals, and graduates are better positioned (Table 5). The categorization of participants’ scores on the ‘perception’ domain has similarities to the previously discussed ‘knowledge and attitude’ domains, except that people over 60 years old scored more ‘Good’ and ‘Fair’ responses (Table 6). The older generation of the population in an earlier study had shown a similar response towards vaccination. The special perception shown by this group of the population could be because, in most pandemics, older generations are classified under the ‘high risk’ category [29].

Although insignificant, around 40% and 22% of respondents did not answer questions on long-term protection offered by vaccines and complications of existing disease post-vaccinations, respectively (Table 4). The apprehensions shown by the respondents could be due to the fact that the precise duration of protection offered by the COVID-19 vaccine is still under research [30,31]. Moreover, the appearance of newer, rare, but fatal adverse events associated with COVID-19 vaccines [26,32] could be the possible reason for participants’ response.

Overall, female participants have better ranking scores for the knowledge, attitude, and perception about COVID-19 vaccination. Previous reports also suggested that women show more information on topics that grossly affect society. The possible reasons identified are education and socioeconomic factors [33,34]. Similarly, people between 18 and 59 years have shown better KAP. This could be because this duration of life is considered to be more productive, and people generally have more curiosity to gain and apply knowledge [35,36].

The participation of both male and female are essential for any survey-based studies. According to the earlier data, women are more likely to take part in the survey studies. However, the participation of females in the present survey was found to be 19% (Table 1). Since both genders of the population are equally responsible for building an opinion on critical issues such as COVID-19 vaccination, identifying the reason for this disparity is important. According to a study, women tend to be more ‘self-select’ in the participation of online surveys than men [37]. Likewise, a study conducted to determine the choice between an online survey and a paper-based survey indicated a gender variation where female participants preferred the paper-based over online surveys [38]. The reasons suggested were that the demographic characteristics of the participating population could play an important role. Factors such as region, ethnicity, educational background, and socioeconomic issues are reported to influence the participants’ choice [37,38]. It can be speculated that one such factor might have contributed to the present study’s discrepancy in gender response. More focused research on this might provide the precise reason for the possible barriers the gender experiences while participating in the online survey studies.

Among nationalities, Saudis showed better KAP, indicating that the participants had updated information about the COVID-19 pandemic and the vaccine that is being used to prevent it. Graduates and above have indicated better knowledge of the COVID-19 vaccine. It is reported that education levels are known to play a positive influence on the current affairs happening around the globe. This reason could be because this group of the population has the urge to gain knowledge, and they analyze the information logically before drawing a conclusion [39].

The Rho value was found to be less than 0.7, suggesting that the responses to domains such as knowledge–attitude, knowledge–perception, and attitude–perception are not correlated. In addition, the significant values were above 0.05, indicating that participants’ responses were not influenced by the three KAP domains (Table 7). Such a type of non-correlated and non-significant association for the participants’ responses has been reported earlier [40]. The finding suggests that survey study respondents independently replied to different questions about KAP domains.

The research on the safety and efficacy is progressing in different parts of the world as the vaccination program is expanded in the population. More reliable information could be available very soon on the precise efficacy and other serious reactions associated with COVID-19 vaccines. Educational programs could be one of the strategies to clarify the apprehensions and create a better society that supports government initiatives to defeat the COVID-19 pandemic [11]. It is essential to remove the social, cultural, and religious misconceptions about the use of vaccines. Hesitancy to vaccines is a common issue found in different parts of the world. Lack of sufficient knowledge and misjudgment influenced by social media are reported to contribute significantly to vaccine hesitancy [10]. Health care providers, in collaboration with government authorities and the media, could remove the misunderstandings of the public. Channels must be created for the effective transfer of transparent scientific information to the general public. Practitioners of the medical profession, community leaders, relatives, and friends must be encouraged to share authentic information about COVID-19 vaccines [41]. Awareness programs conducted by trusted healthcare providers indicating the benefits and important adverse effects of COVID-19 vaccines could build trust in the community [42,43].

The health authorities in Saudi Arabia are working overtime to vaccinate all sections of society. Currently, four COVID-19 vaccines have been approved in the country, such as Pfizer, Moderna, AstraZeneca, and Janssen. These vaccines are available to the public free of cost. In an effort to achieve herd immunity (>70%), the vaccinating centers in the country are open round-the-clock. According to official data, the country is aiming to achieve herd immunity by October 2021, and presently it has inoculated more than 60% of its population. Data from other Gulf states such as the United Arab Emirates (81.6%), Qatar (78.3%), and Bahrain (67.2%) indicated higher percentages of fully vaccinated populations [44]. Considering the geography and the human population of Saudi Arabia, inoculating a significant proportion is a challenging task [44,45]. The data from the present study could augment the efforts of health authorities in reaching their target through designing effective communication strategies for conveying the updated information to the public.

### Limitation of the Study

The study evaluated the responses of only 2022 people residing in Saudi Arabia. The sampling was performed in the month of March–April 2021. The snowball technology used to conduct the study might not have reached the whole population. Some of the responses were not considered due to a lack of complete information. Although best efforts were made to avoid the recall bias and social desirability bias, marginal error in representing the result of the study could not be avoided. Therefore, the opinion expressed in the survey indicates the response of ‘self-selected’ participants with access to the internet within these two months and might not truly reflect the opinion of the country’s whole population.

## 5. Conclusions

This study conducted to determine the knowledge, attitude, and perception of the COVID-19 vaccine indicated that the Saudi population has significant knowledge and a positive attitude. The attitude towards serious unreported adverse reactions and the perception of long-term protection offered by the vaccines has a logical meaning since the available data have not yet established the precise duration of efficacy of the vaccines, and adverse events are not yet linked directly to vaccination. However, the lack of sufficient information about COVID-19 vaccination in pregnancy and children above 12 years needs to be updated. The awareness program must be continued, which has proven to play an important role in communicating information about the safety and efficacy of vaccines to different groups of the population, including those under 18 and pregnant people.

## Figures and Tables

**Table 1:** Demographic characteristics of the participants.

Demographic Variable		Number of Participants (Percentage)
Gender	Male	1638 (81.0)
	Female	384 (19.0)
Age	Less than 18	278 (13.7)
	18–59	1737 (85.9)
	More than 60	7 (0.3)
Nationality	Saudi	1987 (98.3)
	Non-Saudi	35 (1.7)
Educational qualification	Secondary school	101 (5.0)
	High school	650 (32.1)
	Graduation and above	1271 (62.9)

Note: The values are represented as number (Percentage), *n* = 2022.

**Table 2:** Frequency of response for the knowledge questions by the participants.

Questions	Gender	Participant’s Response		
		Yes	No	Do Not Know
Do you have knowledge that Coronavirus infection can be prevented with a vaccine?	Male	1343	95	200
		(82.0)	(5.8)	(12.2)
	Female	317	23	44
		(82.6)	(6.0)	(11.5)
Do you know that the COVID-19 vaccine is available free of cost after registering with the ‘Sahhaty’ App?	Male	1524	39	75
		(93)	(2.4)	(4.6)
	Female	366	13	5
		(95.3)	(3.4)	(1.3)
Are you aware that the COVID-19 vaccine should be taken in two doses?	Male	1398	110	130
		(85.3)	(6.7)	(7.9)
	Female	360	13	11
		(93.8)	(3.4)	(2.9)
Is it true that the administration of the COVID-19 vaccine may cause mild side effects?	Male	1159	88	391
		(70.8)	(5.4)	(23.9)
	Female	326	8	50
		(84.9)	(2.1)	(13)
Is it correct that the COVID-19 vaccine is not recommended for people less than 18 years of age and pregnant women?	Male	695	187	756
		(42.4)	(11.4)	(46.2)
	Female	278	20	86
		(72.4)	(5.2)	(22.4)

Note: The values are represented as number (Percentage), *n* = 2022.

**Table 3:** Frequency of response for the attitude questions by the participants.

Questions	Gender	Participant’s Response		
		Yes	No	Do Not Know
Do you feel that the COVID-19 vaccine must be taken by all whenever it is available?	Male	1036	346	256
		(63.2)	(21.1)	(15.6)
	Female	257	67	60
		(66.9)	(17.4)	(15.6)
Do you think that the COVID-19 vaccine must be given based on priority to a special group of the population, such as older people?	Male	1201	199	238
		(73.3)	(12.1)	(14.5)
	Female	293	44	47
		(76.3)	(11.5)	(12.2)
In your opinion, does the COVID-19 vaccine provide good protection after few weeks against coronavirus infection?	Male	647	276	715
		(39.5)	(16.8)	(43.7)
	Female	141	60	183
		(36.7)	(15.6)	(47.7)
In your understanding, does the COVID-19 vaccine cause any serious reactions apart from what is reported?	Male	405	537	696
		(24.7)	(32.8)	(42.5)
	Female	80	135	169
		(20.8)	(35.2)	(44)
Is it a moral obligation to discuss the benefits and risk factors associated with the COVID-19 vaccine with others?	Male	1059	232	347
		(64.7)	(14.2)	(21.2)
	Female	278	56	50
		(72.4)	(14.6)	(13)

Note: The values are represented as number (Percentage), *n* = 2022.

**Table 4:** Frequency of response for the perception questions by Saudi population.

Questions	Gender	Participant’s Response		
		Yes	No	Do Not Know
Is it essential to vaccinate a large proportion of the population to defeat the coronavirus pandemic?	Male	1174	211	253
		(71.7)	(12.9)	(15.4)
	Female	278	45	61
		(72.4)	(11.7)	(15.9)
Is it important to follow safety measures to avoid COVID-19 even after taking a vaccine?	Male	1228	166	244
		(75)	(10.1)	(14.9)
	Female	333	20	31
		(86.7)	(5.2)	(8.1)
Do you think that the COVID-19 vaccine provides long-term protection against coronavirus infection?	Male	683	359	596
		(41.7)	(21.9)	(36.4)
	Female	128	82	174
		(33.3)	(21.4)	(45.3)
Do you prefer to take the COVID-19 vaccine if you are suffering from any health issues?	Male	568	760	310
		(34.7)	(46.4)	(18.9)
	Female	130	184	70
		(33.9)	(47.9)	(18.2)
Do you prefer to take the COVID-19 vaccine if you are suffering from any health issues?	Male	619	647	372
		(37.8)	(39.5)	(22.7)
	Female	145	154	85
		(37.8)	(40.1)	(22.1)

Note: The values are represented as number (Percentage), *n* = 2022.

**Table 5:** Mean score of participants’ responses on the domains of KAP with respect to demographic characteristics.

Demographic Variable		K-Score	*p*-Value	A-Score	*p*-Value	P-Score	*p*-Value
Gender	Male	3.74 ± 0.02	0.080	3.65 ± 0.03	0.289	3.61 ± 0.22	0.653
	Female	4.29 ± 0.05		3.73 ± 0.02		3.64 ± 0.31	
Age	Less than 18	3.48 ± 0.12	0.101	3.61 ± 0.05	0.273	3.57 ± 0.19	0.749
	18–59	3.90 ± 0.20		3.68 ± 0.11		3.62 ± 0.06	
	More than 60	3.86 ± 0.06		3.00 ± 0.04		3.43 ± 0.04	
Nationality	Saudi	3.85 ± 0.04	0.112	3.89 ± 0.06	0.315	3.62 ± 0.13	0.385
	Non-Saudi	3.54 ± 0.01		3.67 ± 0.07		3.43 ± 0.09	
Educational qualification	Secondary school	3.72 ± 0.08	0.210	3.60 ± 0.06	0.643	3.61 ± 0.17	0.778
	High school	3.72 ± 0.06		3.66 ± 0.05		3.60 ± 0.20	
	Graduation and above	3.92 ± 0.11		3.70 ± 0.05		3.64 ± 0.15	

Note: K-Score = Average knowledge score, A-Score = Average attitude score, and P-Score = Average perception score. Values are expressed as Mean rank ± SD. Statistics: One-way ANOVA and *t*-test followed by appropriate non-parametric tests depending on variables. *n* = 2022; *p* > 0.05 compared within groups.

**Table 6:** Categorization of participants’ scores on KAP domains.

Demographic Variable		N	Knowledge				Attitude				Perception			
		%	Good	Fair	Poor	*p*-Value	Good	Fair	Poor	*p*-Value	Good	Fair	Poor	*p*-Value
Gender	Male	N	1059	364	215	0.110	698	474	466	0.016 *	737	456	445	0.403
		%	64.7	22.2	13.1		42.6	28.9	28.4		45.0	27.8	27.2	
	Female	N	319	52	13		146	140	98		165	143	76	
		%	83.1	13.5	3.4		38.0	36.5	25.5		43.0	37.2	19.8	
Age	Less than 18	N	152	70	56	0.250	116	85	77	0.603	127	82	69	0.599
		%	54.7	25.2	20.1		41.7	30.6	27.7		45.7	29.5	24.8	
	18–59	N	1222	344	171		898	526	313		947	513	277	
		%	70.4	19.8	9.8		51.7	30.3	17.9		54.5	29.5	15.9	
	More than 60	N	4	2	1		3	3	1		2	4	1	
		%	57.1	28.6	14.3		42.9	42.9	14.3		28.6	57.1	14.3	
Nationality	Saudi	N	1358	408	221	0.209	831	605	551	0.466	966	586	435	0.269
		%	68.3	20.5	11.1		41.8	30.4	27.7		48.6	29.5	21.9	
	Non-Saudi	N	20	8	7		13	9	13		15	13	6.4	
		%	57.1	22.9	20.0		37.1	25.7	37.1		44.5	37.1	18.4	
Educational qualification	Secondary school	N	59	26	16	0.002 *	45	31	25	0.376	45	31	25	0.780
		%	58.4	25.7	15.8		44.6	30.7	24.8		44.6	30.7	24.8	
	High school	N	412	155	83		252	212	186		278	203	169	
		%	63.4	23.8	12.8		38.8	32.6	28.6		42.8	31.2	26.0	
	Graduation and above	N	907	235	129		620	371	280		579	365	327	
		%	71.4	18.5	10.1		48.8	29.2	22.1		45.6	28.7	25.7	

Statistics: Chi-square test. *n* = 2022, * *p* < 0.05 compared within groups.

**Table 7:** Correlation between various domains of KAP for participants’ response.

Variables	Rho Value	*p*-Value
Knowledge, Attitude	0.352	0.103
Knowledge, Perception	0.301	0.332
Attitude, Perception	0.548	0.084

Statistics: Spearman’s rho correlation. *p* > 0.05 compared among different groups.

**Table 8:** Multiple stepwise linear regression analysis of factors influencing KAP score.

Independent Variables	Regression Coefficient β	Standard Error	Standardized Regression Coefficient β	*p*-Value
Constant term	17.32	1.009	--	0.000
Age	0.923	0.134	0.189	0.011
Gender	1.231	0.221	0.171	0.032
Nationality	0.453	0.121	0.132	0.062
Level of education	0.212	0.083	0.113	0.091

## Data Availability

Data are contained within the article.
